# Feasibility and acceptability of somatocognitive therapy in the management of women with provoked localized vestibulodynia—ProLoVe feasibility study

**DOI:** 10.1186/s40814-022-01022-2

**Published:** 2022-03-23

**Authors:** Mette Bøymo Kaarbø, Kristine Grimen Danielsen, Gro Killi Haugstad, Anne Lise Ording Helgesen, Slawomir Wojniusz

**Affiliations:** 1 Faculty of Health Sciences, Department of Physiotherapy, Oslo Metropolitan University, Oslo, Norway; 2 Unit for Psychosomatics/CL Outpatient Clinic for Adults, Acute Psychiatric Department, Division of Mental Health and Addiction, Oslo University Hospital, Oslo, Norway; 3 Department of Obstetrics and Gynecology, Oslo University Hospital, Oslo, Norway; 4 Department of Dermatology, Oslo University Hospital, Oslo, Norway; 5 Cognitive Health Research group, Department of Neurology, Division of Clinical Neuroscience, Oslo University Hospital, Oslo, Norway

**Keywords:** Provoked vestibulodynia, Vulvodynia, Vestibulitis, Somatocognitive therapy, Feasibility study

## Abstract

**Background:**

Provoked vestibulodynia (PVD) is a prevalent chronic pain condition especially among young women. Pain is localized to the vulvar vestibule and is provoked by touch or pressure, such as penetrative intercourse. PVD can have profound consequences, adversely affecting a woman’s sexual life, relation to her partner, and her psychological health. There is an urgent need for well-designed randomized clinical trials (RCTs) to identify the most effective interventions for this neglected women’s health condition.

**Aims:**

The primary aim of this study is to assess the feasibility of undertaking a full-scale RCT of somatocognitive therapy (SCT), a multimodal physiotherapy intervention, for women with PVD. The secondary aim is to evaluate the implementation and acceptability of SCT and its potential treatment effectiveness in PVD. In the full-scale RCT, SCT will be compared to standard PVD treatment.

**Methods:**

A multimethod feasibility study with a single-arm before-after trial and qualitative interviews. Ten women with PVD, aged 18–33 were recruited from the Vulva Clinic at Oslo University Hospital. The intervention took place at Oslo Metropolitan University. Participants were assessed at baseline, post-treatment, and the 8-month follow-up with the tampon test and self-report questionnaires. The main feasibility outcomes were evaluation of recruitment rate, adherence to assessment tools, and follow-up rate. The participants’ experiences with the primary outcome and the intervention were explored with semi-structured interviews.

**Results:**

Ten out of 18 eligible patients were recruited over 11 weeks. None were lost to follow-up. Adherence to self-report questionnaires was excellent. Adherence to tampon tests and to the reporting of treatments was good, whereas adherence to the 14-day diary was poor. No adverse events were reported. The tampon test was suboptimal as a primary outcome. SCT was found to be an acceptable treatment, based on Global Perceived Effect scores and the participants’ experiences.

**Conclusion:**

The findings suggest that it is feasible to deliver a full-scale RCT of the SCT intervention for women with PVD. Some changes are suggested to optimize the protocol, such as increasing recruitment sites, change of primary outcome measures, and adding a booster session.

**Trial registration:**

ClinicalTrials.govNCT04208204. Retrospectively registered on December 23, 2019.

**Supplementary Information:**

The online version contains supplementary material available at 10.1186/s40814-022-01022-2.

## Key messages

*What uncertainties existed regarding the feasibility?*

We wanted to investigate the recruitment and follow-up rates, adherence to outcomes, the primary outcome measure, adverse events, and the acceptability of SCT.

*What are the key findings?*

- Follow-up rate, adherence to most outcomes, and acceptability to SCT were judged to be feasible
- The tampon test is suboptimal as a primary outcome in PVD
- The feasibility of the recruitment was below the expected level

*What are the implications of the findings for the design of the main study?*

The findings suggest that it is feasible to deliver full-scale RCT of the SCT intervention in this population, with some adjustments to the protocol:

- Include additional recruitment sites to optimize recruitment
- Replace the tampon test with the Female Sexual Function Index as a primary outcome
- Include a booster session 6 months post-treatment

## Background

Vulvodynia is a multifactorial vulvar pain condition of unknown cause. In the general population, the lifetime prevalence of vulvodynia is estimated between 7 and 16% [1–3] with a higher incidence amongst young women [4, 5]. Although prevalent, vulvodynia is a neglected women’s health condition, where empirically supported treatment guidelines are still lacking [6]. The most common subtype of vulvodynia is provoked vestibulodynia (PVD) [7]. In PVD, pain is localized to the vulvar vestibule and is provoked by touch or pressure such as sexual intercourse and tampon insertion. This pain condition represents the most common cause of painful intercourse [7, 8], adversely affecting women’s quality of life [9–12], psychological health [13, 14], and relation to their partners [15].

The management of PVD is complex and challenging with several treatments available, including pharmacotherapy, surgical, physiotherapy, and psychotherapy. Research evidence, however, is scarce regarding which treatment approach is the most effective. Although physiotherapy is a common first-line treatment for PVD, a systematic review highlighted the need for well-designed randomized controlled trials [16]. Traditionally, physiotherapy treatments for PVD range from internal (vaginal) to external soft tissue mobilizations, joint manipulation, electrotherapy, therapeutic exercises, and pelvic floor exercises [17]. A recent multicenter randomized clinical trial (RCT) also found multimodal physiotherapy to be effective in the management of PVD [18].

Somatocognitive therapy (SCT) is an existing multimodal physiotherapy intervention developed at Oslo University Hospital, Norway, in an attempt to alleviate the burden of longstanding pelvic and gynecological pains [19–21]. In recent years, SCT has been modified and further developed to treat women with PVD, based on experiences from a pilot study [22], from PVD patients [23], and physiotherapy students treating PVD patients [24]. Whereas multimodal physiotherapy treatments are usually provided by physiotherapists specialized in women’s health, SCT is intended to be implemented in primary care and designed to be easy to learn. Furthermore, this approach differs from other forms of physiotherapy for PVD by focusing somewhat less on pelvic floor rehabilitation. SCT is designed to target the multiple dimensions of vulvar pain utilizing a biopsychosocial approach, where the overall aim is to explore and improve body awareness, reduce vulvar pain, and improve sexual function. Other essential components include cognitive strategies to improve coping with negative emotions and thoughts and structured exposure to pain-associated activities. In a recent systematic review on psychosocial factors, a broader approach to PVD was supported [25].

The primary aim of this **pro**voked **lo**calized **ve**stibulodynia (ProLoVe) feasibility study is to assess the feasibility of undertaking a full-scale RCT of the SCT intervention for women with PVD. In the full-scale RCT, SCT will be compared to standard PVD treatment, of which the latter can include women’s health physiotherapy, topical or oral medication, sex therapy, and/or psychological counseling. The main feasibility objectives will evaluate the recruitment rate, the follow-up rate, adherence to the data collection procedure, and number of adverse events. In addition, this study will evaluate the tampon test [26] as a primary outcome measure in preparation for the main trial. The secondary aim is to evaluate the implementation and acceptability of the intervention for the participants and to assess if SCT has the potential to reduce pain, pain catastrophizing, and psychological distress, as well as improve sexual function.

## Methods

### Study design and procedure

This is a multimethod feasibility study with a single-arm before-after trial and qualitative interviews. Ten participants, aged 18–33, were recruited (from February to April 2019) from the Vulva Clinic, Department of Obstetrics and Gynecology, at Oslo University Hospital (OUH)). The SCT intervention took place in the outpatient clinic at the Department of Physiotherapy at Oslo Metropolitan University. The trial consisted of three evaluation points: pre-treatment, post-treatment, and 8 months of follow-up. In addition, the participants were interviewed twice; towards the end of the treatment period and 1 year later.

**Fig. 1:**
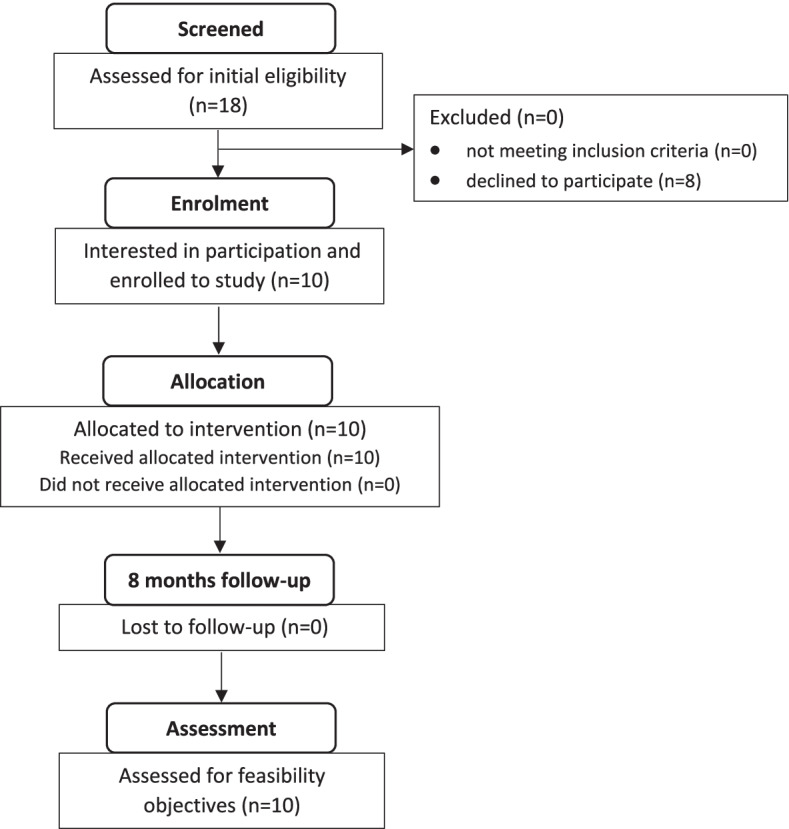
CONSORT 2010 flow diagram. Design and flow of participants through the study

Participant’s eligibility assessment was based on a comprehensive gynecologic examination and medical history, using a standardized protocol for PVD diagnosis [27]. Norwegian-speaking women, aged 18–35, diagnosed with PVD, experiencing pain during (1) penetrative intercourse, (2) pressure applied to the vulvar vestibule, or (3) usage of tampons, were eligible. Patients with an active infection or dermatologic disease in the vulvar region were excluded. Eligible patients were verbally informed about the study and received an information leaflet at the Vulva Clinic. Ten out of 18 eligible women contacted the primary investigator (last author) and received a detailed explanation about the study. All ten agreed to participate. Eight women, however, did not contact the primary investigator and the reasons for this are unknown. The flow of participants through the study is presented in Fig. 1. This study is not intended to be fully powered for the detection of statistically significant effects. The research team therefore decided that ten participants would be an adequate sample size to give a preliminary understanding of the feasibility of undertaking a RCT of the SCT intervention. In the design of the feasibility study, a priority was to interview the participants twice, hence this was one of the reasons we recruited only 10 participants.

### Research ethics

The Regional Committee for Medical and Health Research Ethics in South East Norway (ref. no. 2018/1036, 01.10.18) approved the project. The trial was also registered at ClinicalTrials.gov under the identifier NCT04208204. All participants provided written informed consent prior to participation in this research. The trial is reported according to the Consolidated Standards of Reporting Trial (CONSORT) 2010 statement: extension for pilot/feasibility studies (Additional file 1) [28].

### Data collection

All quantitative data were collected via electronic forms and directly transferred in a secured manner to the Services for Sensitive Data (TSD) research server. Qualitative interviews were recorded with Dictaphone app, which insured immediate and direct transfer of the files to the TSD research server. The qualitative data collected through interviews was transcribed verbatim and uploaded into NVivo 12.

The patients received several electronic assessment packages throughout the study. There were three main assessment time points, baseline, post-treatment, and at 8 months of follow-up.

### Assessment packages at baseline, post-treatment, and 8 months of follow-up

#### Assessment package 1

The participants received an electronic link to the main questionnaire package, which included sociodemographic and clinical characteristics and a battery of self-reported questionnaires such as Female Sexual Function Index [29], Pain Catastrophizing Scale [30], and Hopkins Symptom Check List-25 [31, 32]. The details of these measures are included in Additional file 2. Information about age, number of children, relationship status, education, work status, use of analgesics, body mass index, exercise, and intercourse frequency in the last 4 weeks was collected at baseline.

#### Assessment package 2

The baseline tampon test was undertaken in the evening on days 1, 7, and 14 to measure vulvar pain intensity using the numeric rating scale (NRS) (0–10), where a score of zero represented no pain and 10 meaning the worst possible pain.

#### Assessment package 3

A 14-day diary [33], an *index of emotional instability*, to assess day to day variance in emotional states.

### Registration of received treatment

#### Assessment package 4

Participants received bi-weekly electronic forms up until the 8 months of follow-up recording all treatments received for PVD in the past 14 days. This included all visits to various health professionals, use of medication, and number of sick leave days. In the full-scale RCT, this information will be used to determine what kind of treatments the participants will be receiving and to conduct a cost-effectiveness analysis.

### Tampon test as a primary outcome measure

One of the aims of this feasibility study was to evaluate the tampon test as the primary outcome measure. The tampon test was chosen as a primary outcome based on recommendations for self-report outcome measures in vulvodynia clinical trials [34]. This test has been used as a primary outcome measure in various clinical trials for vulvodynia, evaluating the effect of various treatments [35–41]. It is an alternative measure for pain associated with vulvovaginal penetration and allows the inclusion of women with PVD who are unable to have intercourse. The test has demonstrated good construct validity and reliability [26]. In this study, all women were provided with the same type of tampon as the validity study, the original Regular Tampax^TM^ Tampons [26]. Participants were provided with detailed instructions about how to undertake and record the tampon test, as described by Foster et al. (2009). The participants recorded the degree of pain on the entire tampon insertion and removal experience on the NRS.

### Qualitative interviews

All participants were interviewed one-to-one towards the end of the treatment period. Seven of the women also agreed to take part in a follow-up interview 1 year later. A phenomenological worldview informed the qualitative approach, where the aim was to explore and give voice to the subjects’ perspectives and lived experiences [42]. The second author, a female physiotherapist experienced with qualitative interviews, conducted the interviews and was not involved in the delivery of the treatment. During the first round of interviews, each interview took place in the physiotherapy outpatient clinic at Oslo Metropolitan University. The second interview round was conducted using Zoom, a video meeting platform, due to COVID-19 restrictions. Each interview lasted 60–90 min. A semi-structured interview guide was used to ensure each area of interest was addressed during the interviews, while at the same time encouraging the women to speak freely about their experiences [42]. The interviewer introduced the central topics with open-ended questions, asking the participants to share their experiences with the outcome measures and their experiences with SCT. To elicit rich descriptions, the interviewer tried to follow up salient cues and themes in the participants’ answers, inviting them to elaborate, provide examples, or clarify where appropriate. The interviewer was on the lookout for variations, different angles, and conflicting viewpoints, to promote a nuanced data material [42].

### Intervention

In recent years, SCT has been developed to treat women with PVD [22–24, 43, 44]. SCT is a multimodal physiotherapy treatment approach previously shown to be effective in the treatment of chronic pelvic pain [19–21]. In Table 1, an overview of the intervention is presented as it was provided in this trial, utilizing the template for intervention description and replication TIDierR [45]. The intervention was conducted by the first author, an experienced female physiotherapist trained in SCT.

**Table 1:** Overview of somatocognitive therapy as provided in the feasibility study, as per TIDierR criteria

TIDierR items [45]	Description
**Brief name**	Somatocognitive therapy for provoked localized vestibulodynia (ProLoVe feasibility study)
**Why**	Few RCTs exist, important to develop effective treatments that can easily be applied in primary care. Running a feasibility study is important in preparation for full-scale RCT.
**What**	SCT is a multimodal physiotherapy intervention designed to target the multiple dimensions of vulvar pain, utilizing a biopsychosocial approach. A bodily approach is combined with a cognitive restructuring of negative thoughts. Overall, the aim is to improve body awareness to reduce vulvar pain and psychological distress and improve sexual function.
**Materials: Participants**	Resources: vulva.no
**Materials: Physiotherapist**	Equipment included a treatment bench, mat, pillows, massage balls, mirror, Pilates ball, and educational material.
**Procedures**	Initial appointment: Assess participant—take a thorough history (including previous experiences, beliefs, and expectations) and clinical examination (quality of movement, breathing pattern). The main areas of SCT include the following: ***Therapeutic alliance*** is an essential component of SCT; patient and therapist are in a close working relationship, agreeing on treatment goals and home assignments. Participants take an active part in the decision-making process about their own treatment and progression. ***The bodily approach:*** breathing patterns, maladaptive movement, and postural patterns are addressed in various positions (sitting, standing, walking, and in supine). Through manual techniques and touch, participants are taught various techniques to increase body awareness, improve relaxation, and reduce muscle tension. ***Education*** about PVD, chronic pain, stress, and healthy vulvo–vaginal and sexual behaviors. ***Coping*** with emotions and thoughts related to bodily experiences. Participants learn to become aware of negative/catastrophizing thoughts and learn how to restructure or accept these thoughts as well as how to overcome fear avoidance behavior. An important aspect is the women’s ability to adapt and to self-manage their condition such as coping with pain and flare-ups. ***Structured homework*** promoting the application of learned techniques in daily situations. Gradual exposure to activities associated with pain, desensitization exercises, and exercises to increase the pelvic floor and vulva awareness. Relaxation and breathing exercises. Last session—create a self-management toolbox with participant
**Who provides**	Experienced female physiotherapist trained in SCT, the first author of the article.
**How**	Each session has a three-phased structure: (1) The conversation, (2) the bodily intervention/exploration, and (3) the home assignment.
**Where**	In a closed room with access to the gym, outpatient physiotherapy clinic, Oslo Metropolitan University, Norway Home assignments performed by the participants integrated into ADL
**When and how much**	Initial appointment offered to patients after collection of baseline data. The median number of sessions: 12 (min 7; max 15) face to face with a physiotherapist Treatment period: minimum of 13 weeks and maximum of 22 weeks. Each session (including the initial session) lasted up to 60 min. The number of sessions required was personalized.
**Tailoring**	The treatment is personalized and tailored to the individual. The patient’s participation and collaboration are important. The treatment principles are the same for all but are adapted to suit the individual’s needs.

### Study outcomes

#### Primary feasibility outcomes

The primary aim of this study was to evaluate the feasibility of undertaking a full-scale RCT of SCT for women with PVD. This trial measured several feasibility outcomes in preparation for the main trial. These included the following:

- **Recruitment rate**. This was defined as the number of eligible patients and number of recruited participants per week, within a period of 5 months.
- **The follow-up response rate**. This was measured by the percentage of participants who were followed up successfully until the 8 months of follow-up.
- **Adherence to completion of outcomes**. Adherence was defined as the number of participants who fully completed the battery of self-reported questionnaires, the 14-day diary, the number of performed tampon tests, and biweekly forms about the received treatment, within a time frame of 8 months.
- **Evaluation of the utility value of the tampon test** as a primary outcome measure based on tampon test data and the participants’ experiences with the tampon test (reported in separate mixed methods study [46].)
- **Reporting of adverse events**. Events were recorded as adverse if participants were withdrawn from the study because SCT was deemed as an inappropriate treatment.

#### Secondary outcomes

The secondary aim of this study was to test the implementation and acceptability of the somatocognitive intervention, utilizing both quantitative and qualitative data. The 6-point Global Perceived Effect (GPE) scale was used to provide quantitative estimation of participants’ perceived effect with the treatment directly after treatment and at the 8 months follow-up [47]. The participants were asked “Overall, how much did the treatment you received help your problems?”. The scale ranges from one to six; very much better, much better, a little better, no change, much worse, and very much worse. During the semi-structured interviews, participants were asked about their experiences with SCT intervention, both towards the end of the treatment period and 1 year later. In addition, the aim was to evaluate if SCT has the potential to reduce pain, pain catastrophizing, and psychological distress, as well as the potential to improve sexual function. A description of the self-reported outcome measures is provided in Additional file 2.

### Data analysis

For the feasibility analysis, the results will be expressed as numbers referring to recruitment rate, follow-up, adherence, and adverse events, respectively. Descriptive statistics were used to assess the feasibility objectives and the self-reported outcomes using SPSS (version 27, IBM, Armonk, NY, United States of America) and Microsoft Excel (2016). Due to the nature of a feasibility study and the low number of participants, no hypothesis testing was performed, hence the continuous variables were presented with median and quartile values.

In the qualitative phase, a semantic thematic analysis was performed by the interviewer [48]. Attentive reading and re-reading of the transcripts helped to discern central aspects in the women’s experiences and initial codes were identified. Taking care to include both common and diverging experiences, these initial codes were then reworked and organized into a map of themes and sub-themes, related to experiences with outcome measures, the somatocognitive intervention, and perceived benefits (included in Additional file 3). The first and second author independently reviewed and revised the map for validity against the dataset until an agreement between the authors was reached. The findings are presented as analytical summaries and illustrative quotes, which are fitted under relevant subheadings in the results section. All co-authors took part in the discussion of the final findings.

## Results

Ten nulliparous women with PVD, the median age of 21 (18 to 33), participated in this feasibility study. The sociodemographic and clinical characteristics of the participants are presented in Table 2. Seven of the women reported different comorbidities including jaw pain, muscle pain and twitching, anal pain, endometriosis, headache, migraine, fibromyalgia, irritable bowel syndrome, and alopecia areata. Overall, the women were physically active, exercising from one to three times per week for 30–60 min at moderate to high-intensity levels. Only one reported never exercising. At baseline, four subjects were on oral contraceptives; one on cerazette, one on marvelon, and two on oralcon. In terms of concurrent drug use, all participants reported to have tried topical lidocaine. At baseline, six patients used topical lidocaine on a weekly to daily basis. In addition, one participant was on systemic treatment with amitriptyline, another on levothyroxine, and one on diclofenac.

**Table 2:** Sociodemographic and clinical characteristics of ten women with provoked vestibulodynia

Characteristics	Participants *n*=10
Age *(years)*, median (Q1; Q3)	21 (20; 26)
Pain duration *(years)*, median (Q1; Q3)	7 (3; 8)
Primary PVD	7
Relationship category	
Married/common law	2
In a relationship	2
Single	6
Childbirth	0
Intercourse past 4 weeks	2
Education category	
High school student	1
Undergraduate student	7
Completed bachelor’s degree	2
Work category	
Student	9
Part-time work	5
Full-time work	1
Unemployed	0
Participants with comorbidities	7
BMI, median (Q1; Q3)	23 (20; 23)

### Results of feasibility outcomes

#### Recruitment rate

Eighteen women were found eligible for participation, and ten women contacted the primary investigator, agreeing to take part in the study. Ten participants were recruited over 11 weeks from the Vulva Clinic, achieving a recruitment rate of one participant per week. Recruitment was stopped when the targeted sample of ten participants was reached.

#### Follow-up rate

No participants were lost to follow-up. All the participants completed the SCT intervention, and all partook at all measurement time points up until the 8-month follow-up.

#### Adherence to assessment procedures

Overall adherence to the battery of self-report questionnaires was excellent, with all participants completing all the self-reported questionnaires at all three time points. In terms of adherence to the tampon tests across the three measurement time points, 81 out of 90 tampon tests were completed (90%). At baseline, there were nine full tampon test sets. At post-treatment, there were eight full tampon test sets and at the 8-month follow-up, there were six full tampon test sets. For the 14-day diary, there were six full data sets at baseline, three post-treatment, and two at the 8-month follow-up. Regarding adherence to the bi-weekly forms about received treatments, two women did not record any of the SCT treatments; however, all the other treatments received were recorded.

### Evaluation of the tampon test as the primary outcome measure

Evaluation of the tampon test is reported in a separate paper [46]. We concluded that the test may be suboptimal as a primary outcome measure in PVD research. The tampon test data demonstrated large intra- and inter-individual variability; furthermore, the test seems to underestimate the severity of pain in some women with PVD. Out of ten women with PVD, four of the women had an NRS score that was equal to, or below four, whilst concurrently reporting high levels of pain during sexual intercourse. Participants with low pain scores would be excluded from studies where the tampon test is part of the trial eligibility criteria, even though severe pain was experienced during sexual intercourse. Several women also reported in the interviews that they experienced the test as an inadequate measure of their problem [46].

#### Reporting of adverse events

There were no adverse events reported, that is no participants were withdrawn from the study because SCT was considered as an inappropriate treatment. All participants turned up for their scheduled appointments and completed the intervention.

### Results of secondary outcomes

#### Implementation and acceptability of somatocognitive therapy

In this study, the participants received a median number of 12 sessions. The SCT was personalized, hence the number of treatments delivered varied from seven up to a maximum of 15 sessions. In the original protocol, we stipulated that treatment duration would last for up to ten weeks. In this study, the treatment course lasted a minimum of 13 weeks and a maximum of 22 weeks. The frequency of the treatment delivery varied as it was personalized to the individuals’ needs. Patients communicated to the therapist that they needed time to practice home assignments and incorporate what they had learned into their ADL. The frequency of treatment was also influenced by external factors, such as study and work commitments, exams, and holidays.

Half of the participants were content with the number of treatment sessions received and felt ready to continue by themselves when the treatment period ended. The other half reported that they would have preferred a slightly longer treatment period. Several found it more difficult to keep motivated to prioritize their recovery process when their progress was no longer monitored by the therapist. *P8:* “Immediately after the treatment period ended it felt a bit tough. You receive such close guidance, and then you are suddenly alone with it again. I found it a bit difficult to keep my motivation up”. Most felt they would have benefited from one or two follow-up sessions a few months later, for repetition, motivation, and guidance on how to move forward. *P3:* “Perhaps it could have been possible with a follow-up session six months later, in case things should get worse or you need some repetition, or when things have just been a little too much.”

#### Participants’ perceived effect of SCT

The GPE scale was used to provide quantitative estimation of participants’ perceived effect of the treatment measured directly after treatment and at 8 months of follow-up. Directly after treatment, three women reported to be *very much better*, four *much better*, and three *a little better*. At 8 months of follow-up, one participant reported to be *very much better*, six participants reported *much better*, two reported *a little better*, and one reported *no change*.

#### Participants’ experiences with the intervention

All participants expressed positive experiences with the SCT approach. They found it useful to learn techniques for deep breathing, relaxation, and self-management, as well as developing more constructive ways of thinking about and relating to their pain and sexuality. The participants largely expressed beliefs that PVD is complex and multidimensional in nature. They found it meaningful to combine physical and psychological aspects and not exclusively focus on the painful vulvar area. *P3:* “I feel that somatocognitive therapy is more focused on the long-term recovery process. That it is easier to get lasting results when you not only treat the local muscles or problem area, but also include everything else around”. Furthermore, the importance of taking responsibility for their own recovery process was expressed by P6: “It makes so much sense that this is what I have to do. Not just talk about it and not just receive massage here or there. I have to make an active effort. Breathe. I have to relax”. Three participants however (P2, P7, and P9), felt the intervention would have benefitted from a specific focus on the vulvar area, including manual techniques to release tensions in the pelvic floor muscles. Most of the participants however appreciated the gentle and desensitizing approach to the vulva. Several women also expressed that the encouragement to explore their own vulvas had helped them develop a more positive way of relating to this area. *P4*: I feel like I have made great improvements, as before my vulva felt very unfamiliar, I just didn´t want to think about it. But now I actually feel that I have developed a completely different way of thinking about it and how it also is about being less afraid of the area”.

The secondary aim of this study was to evaluate if SCT intervention had the potential to improve sexual function and reduce pain, pain catastrophizing, and psychological distress. The women improved on all the outcome measures from baseline to post-treatment, with a slight deterioration of the effect at the 8-month follow-up. Table 3 includes all the measurements and number of participants who had experienced intercourse in the past 4 weeks, at the three time points.

**Table 3:** Measurements at baseline, post-treatment, and 8 months of follow-up (*n*=10), (none lost to follow-up)

	Pre-treatment	Post-treatment	8 months of follow-up
**Tampon test** NRS (0-10), median (Q1; Q3)	4.5 (2.5; 6)	2 (1.5; 4.2)	3.5 (1.8; 4.5)
Intercourse past 4 weeks, n	2	6	7
**FSFI**, median (Q1; Q3)			
Total sum (0–36)	14.8 (9.8; 19.8)	22.8 (15.8; 25.4)	20.9 (18; 27.1)
Desire	2.1 (1.6; 3.2)	3.6 (2.3; 3.8)	3.6 (2.7; 4.3)
Arousal	3.2 (1.6; 4.9)	4.4 (2.7; 5.7)	4.4 (3.0; 5.6)
Lubrication	4.2 (2.9; 5.2)	4.8 (3.5; 5.8)	4.7 (3.6; 6.0)
Orgasm	3.2 (0.9; 5.3)	4.8 (2.6; 5.3)	4.8 (1.2; 5.2)
Satisfaction	0.8 (0.4; 2.0)	4.2 (1.1; 5.2)	3.8 (1.2; 5.3)
Pain	0.0 (0.0; 0.3)	1.8 (0.0; 3.6)	2.0 (0.0; 3.6)
**PCS (0–52)**, median (Q1; Q3)	20 (15.3; 29.3)	9.5 (5.3; 20)	12.5 (6.3; 22)
**HSCL-25**, median (Q1; Q3)	2.0 (1.7; 2.5)	1.6 (1.3; 2.4)	1.8 (1.6; 2.2)

*NRS* Numerical Rating Scale (higher scores indicate more pain), *FSFI* Female Sexual Function Index (higher scores indicate better sexual function), *PCS* Pain Catastrophizing Scale (higher scores indicate higher levels of catastrophizing, *HSCL-25* Hopkins Symptom Check List - 25 (higher scores indicate higher levels of psychological distress)

## Discussion

This study was designed to assess the feasibility of running a full-scale RCT of the SCT intervention for women with PVD. In addition, the implementation and acceptability of SCT were evaluated, including its potential as a treatment for PVD. The current study demonstrated that the study was feasible with respect to follow-up rate and adherence to the assessment outcomes. We would argue that the intervention was acceptable based on Global Perceived Effect scores, the participants’ experiences with the intervention, and the changes seen on the outcome measures. No adverse effects were reported. Based on the feasibility findings, a few changes are suggested to optimize the protocol. In the following section, the feasibility outcomes and secondary outcomes will be interpreted and further discussed.

In terms of recruitment for the study, ten out of 18 patients were recruited over 11 weeks from one site, achieving a recruitment rate of one participant per week. In the planned future RCT, we aim to recruit 130 patients. Power analysis suggests that 128 participants, split equally between the study arms, will be enough to show the between-group difference in the total score on the primary outcome, Female Sexual Function Index (FSFI), at the 12 months of follow-up of at least three points (sd = 6.0). We will use *α* = 0.05 and 1-β = 0.8. In this feasibility study, we recorded an average improvement of six points on the FSFI in the course of 8 months in patients treated with SCT. To run a fully powered RCT with 130 patients, recruited in approximately 24 months, we will have to expand recruitment sites to other gynecologists experienced with PVD located at various clinics in the Oslo area.

Feasibility outcomes related to follow-up rate and adherence were overall satisfactory. No participants were lost to follow-up, and adherence to completion of the battery of self-reported questionnaires was excellent, with all ten participants fully completing the questionnaires. We demonstrated good adherence to the tampon test, with 81 out of 90 tampon tests completed (90%). Most of the missing tampon test data occurred at 8 months of follow-up. The 14-day diary, however, the *Index of emotional instability*, which was used to measure day-to-day variance in emotional states, had high levels of missing data. At the 8-month follow-up, there were only two complete 14-day diary sets, hence the diary will not be included in the main trial. During the interviews, many women found the diary time-consuming and difficult to remember, and for some, it also felt irrelevant. Adherence to the reporting of received treatment was satisfactory with eight full sets at the 8-month follow-up. Two women did not record any of the physiotherapy treatments received, possibly due to a misunderstanding as all the other treatments they had received had been recorded.

A further aim with this study was also to evaluate the feasibility of using the tampon test as a primary outcome measure. Many women with PVD abstain from penetrative intercourse and have difficulties with reporting pain. The tampon test was therefore chosen as a primary outcome measure as it was specifically designed to address this challenge [26]. Based on the tampon test data and the participants’ experiences with the test and input from a user representative, we concluded in a separate paper [46] that the tampon test is suboptimal as a primary outcome measure in PVD research. Therefore, in the upcoming RCT, the primary outcome measure will be the Female Sexual Function Index (FSFI), while the tampon test will be applied as a secondary outcome. FSFI is widely used in PVD research [18, 49, 50]. In addition to pain experienced during intercourse, it captures several other dimensions of sexual functioning. In this study, the participants improved on average by 7.9 points (from 14.8 to 22.7 post-treatment) and 6.1 points (from 14.8 to 20.9) at the 8-month follow-up, when accounting for all subscales on the FSFI. We observed a very similar magnitude of changes on all subscales, which conforms to the notion of a multidimensional nature of this disorder. In preparation for the upcoming main trial, the choice and implementation of outcome measures, including the FSFI, will be based on the findings from this feasibility study and *Recommendations for the study of vulvar pain in women, part I: review of assessment tools* [51].

Overall, we would argue that SCT is an acceptable and promising intervention. These findings are in line with previous studies evaluating the effect of SCT for women with chronic pelvic pain [19–21] and women with PVD [22]. SCT is designed as a short-term therapy where one of the goals is to promote self-management of PVD and avoid over-treatment and therapist-dependency. Although approximately half the women were satisfied with the amount of treatment received, some expressed that the treatment ended too soon and described how a booster session would be valuable. This would provide an opportunity to receive support and guidance over time. Consequently, in the future RCT, the participants will be offered one booster session of 6 months after the end of the treatment.

Participants’ perceived effect was measured with the GPE and was further supported by the qualitative interviews, with most women reporting a variety of improvements following the SCT intervention. In terms of pain reduction, most women described a recovery process characterized by periods of improvements and setbacks, but overall experienced a positive development. Meaningful changes also included improved body awareness; an improved ability to relax, feel more connected, and be comfortable in their own bodies. Several of the participants also described how the intervention had helped them develop a more neutral and less fearful way of thinking about their pain, which was also supported by the findings on the pain catastrophizing scale. Furthermore, the women had gained more healthy attitudes and strategies regarding their sexuality and some felt more confident involving their romantic partner in the recovery process.

This study allows us tentatively to assess the effectiveness of the intervention in a small sample of participants. This study was not powered to detect changes over time and results should therefore be interpreted with caution. Albeit, all outcome measures pointed in the same direction, as the participants improved on the primary outcome, i.e., the tampon test, and all the secondary outcomes in the main questionnaire packet following SCT. The results indicate that the intervention has potential treatment effectiveness in a small sample of participants with PVD.

A limitation of this study was the small sample size, as it is challenging to compute precision around estimates for recruitment, follow-up, and adherence with such small numbers. A further limitation of this study was the lack of an active control group. In the main trial, SCT will be compared to standard treatment, hence the design of this study does not match the design of the future study. We therefore lack information regarding the participants’ willingness to be randomized to either the intervention or the control group. A control group could also have provided us with valuable information about the treatments delivered in this group, as well as the feasibility of the randomization process, including the follow-up rate. The strength of this study is that both quantitative and qualitative methods were implemented to evaluate the feasibility and acceptability of SCT.

## Conclusion

We conclude that it is feasible and practical to deliver a RCT of SCT, a multimodal physiotherapy intervention, in women with PVD. The aims of the feasibility study have been met. Some changes, however, are suggested to optimize the study protocol, before conducting a full-scale RCT. This includes replacing the tampon test with the FSFI, increasing the recruitment sites, and adding a booster session.

## Supplementary Information

**Additional file 1.** Consort 2010 checklist.

**Additional file 2.** Patient reported outcome measures.

**Additional file 3.** An overview of the analytical process, moving from the preliminary themes to the main theme.

## Data Availability

Anonymized individual-patient datasets are available from the corresponding author on reasonable request.

## Abbreviations

FSFI: Female Sexual Function Index
HSCL-25: Hopkins Symptom Check List – 25
NRS: Numeric Rating Scale
PCS: Pain Catastrophizing Scale
PVD: Provoked vestibulodynia
RCT: Randomized clinical trial
SCT: Somatocognitive therapy
