# Supplementary material for: Feasibility and acceptability of somatocognitive therapy in the management of women with provoked localized vestibulodynia—ProLoVe feasibility study
Source: Pilot Feasibility Stud. 2022 Mar 23;8:68. doi: 10.1186/s40814-022-01022-2 (PMC8941371; doi:10.1186/s40814-022-01022-2)
Supplement: Supplementary file 2 — Additional file 2. Patient reported outcome measures. [file 40814_2022_1022_MOESM2_ESM.docx]

**Additional file 2** Patient reported outcome measures

**Tampon test:** The tampon test is an alternative measure for vulvovaginal penetration and allows the inclusion of women with PVD who are unable to have intercourse. The test has demonstrated good construct validity and reliability (31). Degree of pain is recorded on the entire tampon insertion and removal experience on the Numerical Rating Scale (NRS) (0-10), where a score of zero represented no pain and 10 meaning the worst possible pain. The test is undertaken three times, on day 1, 7 and 14.

**The Female Sexual Function Index -19** (FSFI) is a 19-item patient-reported outcome measure (34) extensively used in clinical trials to measure female sexual function. FSFI is commonly used in PVD research (24, 54, 55) and is recommended in the *Recommendations for the study of the vulvar pain in women, part I: review of assessment tools* (56). FSFI consists of 6 separate domains of female sexual function, namely desire (items 1-2), arousal (3-6), lubrication (7-8), orgasm (11-13), satisfaction (14-16), and pain (17-19). Higher scores are indicative of higher levels of sexual functioning.

**The Pain Catastrophizing Scale** (PCS) was introduced in 1995 (35). It is a self-report measure, consisting of 13 items about thoughts and feelings related to pain. Answers are given on a 5 point Likert scale from 0 (not at all) to 4 (to all the time), resulting in a total possible score of 52. Higher scores indicate greater levels of catastrophizing. The PCS is broken into three subscales being rumination, magnification and helplessness. The Norwegian PCS demonstrates acceptable psychometric properties when applied to patients with low back pain (57).

**The Hopkins Symptom Checklist-25** (HSCL-25) is a self-report measure of psychological distress, proved to have satisfactory validity and reliability (36), commonly used in Norway. The questionnaire consists of 25 questions about anxiety, depression and somatization. HSCL-25 is a shorter version of the Symptom Checklist 90 (SCL-90) and consists of 25 items that are rated from 1 (not at all) to 4 (extremely). The score will be obtained by averaging the scores. A higher average score is indicative of higher levels of psychological distress. For the total score, an average cut-off point 1.75 is a valid predictor of mental disorder (58).

**Global Perceived Effect scale (GPE)** The patient rated perception of change post treatment and at eight months follow-up was assessed on a 6-point Global Perceived Effect scale (GPE). (52). The participants were asked “overall, how much did the treatment you received help your problems”. The scale ranges from one to six. The responses were: Very much better, much better, a little better, no change, much worse and very much worse.
