# Supplementary material for: Feasibility and acceptability of somatocognitive therapy in the management of women with provoked localized vestibulodynia—ProLoVe feasibility study
Source: Pilot Feasibility Stud. 2022 Mar 23;8:68. doi: 10.1186/s40814-022-01022-2 (PMC8941371; doi:10.1186/s40814-022-01022-2)
Supplement: Supplementary file 3 — Additional file 3. An overview of the analytical process, moving from the preliminary themes to the main theme. [file 40814_2022_1022_MOESM3_ESM.docx]

**Additional file 3** An overview of the analytical process, moving from the preliminary themes to the main theme.

| **Step 1 – Codes** | **Step 2 – Subthemes** | **Step 3 - Themes** |
| --- | --- | --- |
| Individual needs regarding session frequency and length of therapy  Important with flexibility and tailoring  Challenging to adjust from close follow-up to complete independency  Experiencing ups and downs  Difficult keeping up motivation after end of therapy  Easy to slip back into old habits  Booster session could be useful | Valuable that intervention dose and frequency is tailored to the individual  Need for booster session | SCT implementation |
| Meaningful with a whole person approach  Valuable to understand and experience how mind and body are connected  Useful to learn techniques to handle pain, but also life in general, better  Helpful with a gradual and desensitizing approach to the vulva, however some miss a more hands-on approach  Pain reduction  Improved awareness of and connection to the body  More comfortable in the body  Less fearful of vulva  Better able to relax  A new way of relating to the pain  A more positive inner dialogue | A meaningful and educational approach  Positive impact and change | SCT meaning and perceived benefits |
